# Effect of Waste Glass on the Properties and Microstructure of Magnesium Potassium Phosphate Cement

**DOI:** 10.3390/ma14082073

**Published:** 2021-04-20

**Authors:** Qiubai Deng, Zhenyu Lai, Rui Xiao, Jie Wu, Mengliang Liu, Zhongyuan Lu, Shuzhen Lv

**Affiliations:** State Key Laboratory of Environmental-Friendly Energy Materials, School of Materials Science and Engineering, Southwest University of Science and Technology, Mianyang 621010, Sichuan, China; qiubaiswust@163.com (Q.D.); x929988907@163.com (R.X.); wujie_0828@163.com (J.W.); guyuaoxiang@163.com (M.L.); Luy@swust.edu.cn (Z.L.); Lvshuzhen@swust.edu.cn (S.L.)

**Keywords:** magnesium potassium phosphate cement, glass powder, hydration process, microstructure

## Abstract

Waste glass is a bulk solid waste, and its utilization is of great consequence for environmental protection; the application of waste glass to magnesium phosphate cement can also play a prominent role in its recycling. The purpose of this study is to evaluate the effect of glass powder (GP) on the mechanical and working properties of magnesium potassium phosphate cement (MKPC). Moreover, a 40mm × 40mm × 40mm mold was used in this experiment, the workability, setting time, strength, hydration heat release, porosity, and microstructure of the specimens were evaluated. The results indicated that the addition of glass powder prolonged the setting time of MKPC, reduced the workability of the matrix, and effectively lowered the hydration heat of the MKPC. Compared to an M/P ratio (MgO/KH_2_PO_4_ mass ratio) of 1:1, the workability of the MKPC with M/P ratios of 2:1 and 3:1 was reduced by 1% and 2.1%, respectively, and the peak hydration temperatures were reduced by 0.5% and 14.6%, respectively. The compressive strength of MKPC increased with an increase in the glass powder content at the M/P ratio of 1:1, and the addition of glass powder reduced the porosity of the matrix, effectively increased the yield of struvite-K, and affected the morphology of the hydration products. With an increase in the M/P ratio, the struvite-K content decreased, many tiny pores were more prevalent on the surface of the matrix, and the bonding integrity between the MKPC was weakened, thereby reducing the compressive strength of the matrix. At less than 40 wt.% glass powder content, the performance of MKPC improved at an M/P ratio of 1:1. In general, the addition of glass powders improved the mechanical properties of MKPC and reduced the heat of hydration.

## 1. Introduction

According to a United Nations Environment Programme survey, 1.4 billion tons of waste glass is discarded each year, including flat glass, daily glassware, and glass packaging containers, accounting for more than 80% of glass products [1]. Most of these waste glasses consist of broken glass and substandard glass in production, and the random disposal of these glasses can also cause great harm to the environment [2]. Therefore, recycling waste glass is critical.

To improve the recycling of waste glass, many countries have established recycling policies. The United States has classified glass chips and bottles as environmental pollutants that must be recovered [3]. Italy has issued a law specifically addressing the recycling of beverage bottles [4]. Japan has more than 200 waste glass recycling centers, with a recycling rate of approximately 50% [5]. At present, glass is typically recycled in one of four ways [6]: (1) as a casting solvent, (2) as cullet to reduce energy consumption during new glass manufacturing, (3) reuse of raw materials, and (4) conversion and utilization. The performance of concrete can be improved by adding admixture to building materials. The studies of some scholars show that polypropylene fiber and silica fume [7,8], untreated coal and recycled aggregate [9], and untreated coal gangue [10] can improve the performance of concrete. For the application and development of waste glass, as early as the 1960s, attempts were made to apply waste glass in concrete [11,12], replacing fine aggregates. Unfortunately, adding glass to concrete reduced its strength due to the alkali-silica reaction (ASR), hindering its utilization. Later, waste glass was ground into a powder form and applied as an auxiliary cementitious material in cement and concrete [13], which enhanced the mechanical properties and microstructure of the cement and concrete, improved their basic chemical properties, and increased their durability. Therefore, the application of waste glass to building materials is of great interest to researchers, engineers, and builders in the construction industry.

In recent years, many researchers have investigated a new type of cement called magnesium phosphate cement (MPC), because of its many advantages, including fast setting and hardening [14], high early strength [15], high-temperature resistance [16], low shrinkage [17], and strong adhesion [18]. However, because of its high cost and a series of engineering problems, MPC requires further research and development before it can be widely applied. The main hydration reaction of MPC is an acid-based neutralization of dead-burned MgO and soluble phosphate, and the solution-diffusion mechanism has been recognized by many scholars [19]. Hydration is mainly divided into three stages [20]: (1) phosphate dissolution in a liquid environment forms PO_4_^3−^, H^+^, and HPO_4_^2−^ plasma, and in a weakly acidic environment, hydration promotes the dissolution of MgO, where the Mg^2+^ develops a positively charged hydrosol, Mg(H_2_O)_n_^2+^; (2) the hydrosol reacts with the phosphate ions in the system to form hydration products, and the hydration products are connected; (3) the later stage of hydration will form a network structure with MgO as the skeleton and hydration products as the adhesive.

Nevertheless, a series of problems are encountered, both in the current research and in engineering applications of MPC, including the high cost of raw materials, rapid setting time, high exothermic hydration of MPC, and the lack of relevant international standards. MPC performance was effectively improved by adding an admixture to the MPC components, thereby reducing the demand for dead-burned MgO. For example, metakaolin effectively improves the MPC compressive strength, tensile bond strength, freeze-thaw resistance, and water resistance [21]; glass fiber significantly improves the MPC early strength and enhances the flexural strength of the mortar [22]; Fe_2_O_3_ can promote the crystallization of Struvite-K, prolong the setting time of the slurry, and reduce the flow rate [23]; microsteel fiber (MSF,) polyvinyl alcohol (PVA) fiber, and basalt fiber (BF) improve the flexural parameters (toughness coefficient, toughness index, and equivalent flexural strength ratio) [24]; and water glass shortens the setting time, enhances the compressive strength of the hardened mortar, and accelerates the increase in the slurry viscosity [25]. Waste glass can also be used in magnesium phosphate cement. Although its chemical stability hinders its reactivity in weakly acidic and neutral environments [26]. However, in MPC, the rapid release of the hydration heat and the weak alkaline environment in the hardening stage may promote a chemical reaction with the glass powder.

Meanwhile, replacing MgO with an admixture of glass powder reduces the price of the raw materials. In this regard, some scholars have studied the addition of glass powder to magnesium ammonia phosphate cement (MAPC). The results indicate that the alkali component of glass powder (GP) is involved in the MAPC reaction. The addition of GP to MAPC promotes the crystallization process of the hydrate and produces secondary reaction products. When 10 wt.% glass powder is added to a magnesium-to-phosphate (M/P) ratio of 3:1, the matrix’s workability and setting time meet the rapid repair material requirements, and the matrix has a denser microstructure, thereby improving the compressive strength [27]. However, the effects of higher concentrations of GP combined with various M/P ratios on the hydration heat release are unknown. Therefore, this study intends to evaluate the variation in GP content at variable M/P ratios on the workability, setting time, strength, hydration heat release, porosity, and microstructure of magnesium potassium phosphate cement (MKPC).

In this study, waste glass was ground into powder and added to MKPC to compensate for its limitation of application as a rapid repair cement. First, glass powder was substituted for a fraction of the dead-burned MgO, and the workability, setting time, and hydration temperature rise of the slurry at various M/P ratios were investigated. Next, the compressive strength of the samples was evaluated, and the porosity of the samples after curing for 28 d was determined by the piezometric porosity method. The type and yield of the hydration products were identified using X-ray diffraction (XRD) and thermogravimetric analysis. Finally, the morphological structure of the hydration products was examined by scanning electron microscopy and energy dispersive spectroscopy.

## 2. Materials and Methods

### 2.1. Experimental Material

**Figure 1:**
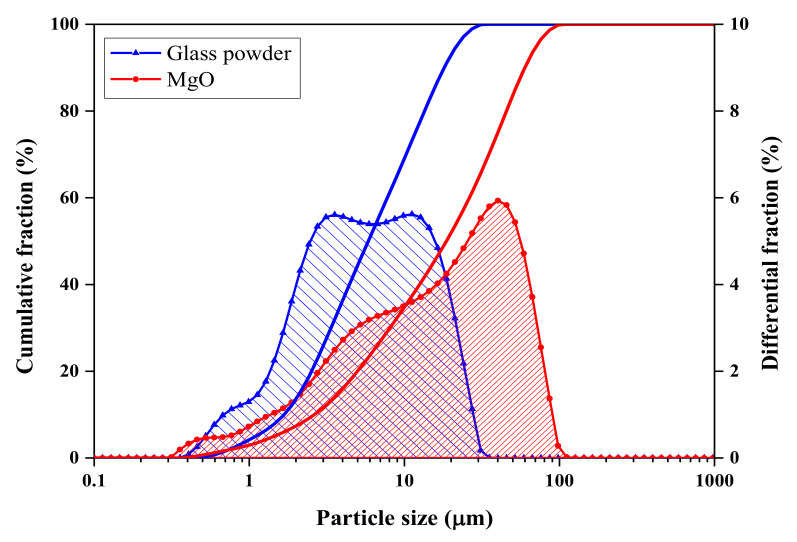
Particle size distribution of dead-burnt MgO and glass powder.

The raw materials used in this experiment included dead-burnt MgO (Yancheng Huanai Magnesium Industry Co., Ltd., Jiangsu, China, industrial purity, 95%). The MgO particle size distribution and chemical composition are shown in Figure 1 and Table 1. The second raw material component was potassium dihydrogen phosphate (KH_2_PO_4_, referred to as (KDP), analysis reagent, > 99.0%) produced by the Chengdu Jinshan Chemical Plant, China. Borax (Na_2_B_4_O_7_·10H_2_O, referred to as B, analysis reagent, > 99.5%) produced by the Chengdu Cologne Chemical Plant, China, was the third component. Glass was ground into glass powder from waste glass, and the chemical composition and particle size distribution are shown in Figure 1 and Table 1.

### 2.2. Mixture Design

In this experiment, the effects of different glass powder contents (0 wt.%, 10 wt.%, 20 wt.%, and 40 wt.%) on the hydration and hardening properties of MKPC with M/P ratios (MgO/KH_2_PO_4_ mass ratio) of 1:1, 2:1, and 3:1 (MgO/KH_2_PO_4_ molar ratio of 3.4:1, 6.8:1 and 10.2:1) were studied. The details are presented in Table 2.

Based on the experimental design, MgO, KDP, B, and glass powder were mixed first, and then the dry powders were stirred for 1 min. Next, water was added and slowly stirred for 30 s, quickly stirred for 30 s, stopped for 30 s, and finally stirred for another 90 s, ensuring that all raw materials were thoroughly mixed. The stirred slurry was poured into a 40 mm × 40 mm × 40 mm mold and vibrated for 60 s on the cement mortar vibration table. After demolding, the sample was held at 20 ± 1 °C at 60 ± 5% humidity for 1, 3, 7, and 28 d.

### 2.3. Analysis and Characterization Methods

The particle size distribution of MgO and the glass powder was analyzed using a Mastersizer 2000 (Malvern, UK). The particle sizes of MgO and glass powder were tested in anhydrous ethanol solution and ultra-pure water solution, respectively. The compressive strength of the sample was tested using a microcomputer-controlled electro-hydraulic servo pressure testing machine (HCT-605A, Shenzhen Wanchao Test Equipment Co., Ltd., Shenzhen, China). The main engine employed a high-performance bi-directional hydraulic cylinder loading with high reliability, zero leakage, a maximum load of 3000 kN, and a loading speed of 2.4 kN/s. The chemical composition of the sample was analyzed by an X-ray fluorescence spectrometer (Model: Axios; PANalytical B.V., Almelo, The Netherlands; main technical specifications: maximum power 2.4 kW; angle reproducibility better than ±0.0001°; accuracy 0.0025°; the detector is scintillation detector and flow detector; analytical element range is 9F-92U, the content range is 0.01–100%; sample diameter is 32 mm).

The setting time was determined according to the Chinese standard method specified by JGJ/T 70-2009 [28] and began when the sample came into contact with water in the mixing pot, as determined by the Vika standard instrument produced by Shaoxing Xingsheng Instrument Co., Ltd., Shaoxing, China. Owing to the rapid hydration rate of MKPC, samples were only tested after reaching the final setting time. The workability was measured according to the Chinese standard method specified in GB/T 2419-2005 [29]. After lifting the truncated cone die vertically and gently upward, the mixture flowed freely (mixtures were not placed on the vibration table) for approximately 30 ± 1 s. The diameters of the two directions perpendicular to each other on the bottom of the mixture were measured with a caliper and the average value in millimeters was calculated.

The pore size distribution, total pore volume, total pore area, and other physical properties of the sample were measured using an Autopore 9500 mercury porosimeter (Micromeritics Company, Atlanta, GA, USA). The maximum pressure was 33,000 lb (228 MPa), and the aperture analysis range was 5.5 nm to ~360 µm.

The samples were ground into powders in an agate mortar and then analyzed using a DMAX1400 X-ray diffractometer (scanning speed 0.333°2/s, scanning range 10–40°) made by Rigaku, Tokyo, Japan. The morphology and micro-area composition of the hydration products of the solidified body were tested by a Variable temperature in situ imaging analysis system (model TM4000, made by Hitachi High-Technologies Corporation, Shanghai, Acceleration voltage: 5 kV, 10 kV, 15 kV, 20 kV; sample movable range: X: 40 mm Y: 35 mm; vacuum mode: standard and electrostatic reduction; image signal: backscattered electrons.). The samples are gilded with a small ion sputtering apparatus (SBC-12, Beijing, China). Differential thermal analysis of the MKPC-hardened body was performed using a thermal analyzer (STA8000, Perkin–Elmer, Waltham, MA, USA). The heating rate was 20 °C/min, and the test temperature range was 30–900 °C.

## 3. Results and Discussion

### 3.1. Workability and Setting Time

**Figure 2:**
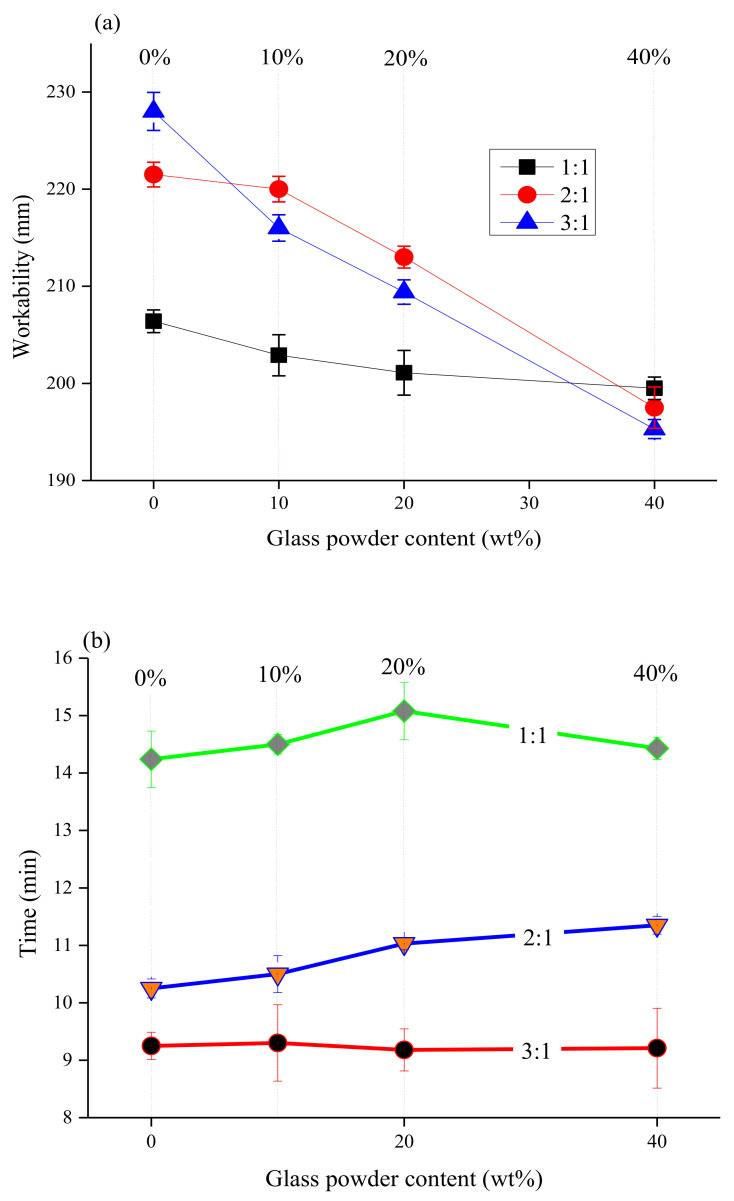
Setting time and workability of the MKPC specimens as a function of glass powder content (0, 10, 20, and 40 wt.%) and M/P ratio (1:1, 2:1, and 3:1), (**a**) Setting time; (**b**) workability.

Figure 2 shows the effect of glass powder content (0 wt.%, 10 wt.%, 20 wt.%, and 40 wt.%) on the workability and setting time of MKPC paste with different M/P ratios (1:1, 2:1, and 3:1). In Figure 2a, with an increase in the glass powder content, the workability of the MKPC pastes decreased gradually, and the workability of G1-4 was 3.3% lower than that of G1-0. Compared with G2-0 and G3-0 without glass powder, the workability of G2-4 and G3-4 decreased by 10.8% and 14.3%, respectively. Glass powder was added to the system as a replacement for MgO, because of its non-absorbent surface and acid resistance [2], and it did not participate in the hydration of MKPC paste in the early reaction. The particle size of the glass powder was mainly concentrated in the range of 1–30 µm, making it an ultra-fine glass powder [30]. The influence of glass powder on the workability of the paste is affected by the following factors [31]: the shape and size of the particles, the lack of water absorption of the glass, the smooth surface of the glass particles, the influence of the wettability of the glass particles, and the amount of cement replacement. The lack of water absorption of glass particles increases the effective water-to-cement ratio (w/c) and enhances workability. However, the shape of the glass powder largely determines the workability of the paste, where particles with an irregular shape, a large aspect ratio, and angular or flake-like particles reduce the workability. When the M/P ratio increases, there is a large amount of unreacted MgO cement in the matrix, which increases the frictional force of the particles. Thus, a mixture with a high M/P ratio is more viscous.

In Figure 2b, the addition of glass powder can delay the setting time of the MKPC paste. When the glass powders were not added, the setting times of G1-0 and G2-0 were 14 min 20 s and 10 min 25 s, respectively. When the content of the glass powder was 20 wt.%, the setting time reached 15 min 10 s and 11 min 10 s for G1-2 and G2-2, respectively, indicating that the addition of glass powder delayed the setting time of the MKPC paste. The addition of glass powder reduced the content of active MgO in the system, affecting the hydration reaction of the MKPC, and slowing the setting time of the slurry. The higher content of MgO minimized the effect of the glass powder on the setting time of the MKPC paste.

### 3.2. Heat Release during Hardening

**Figure 3:**
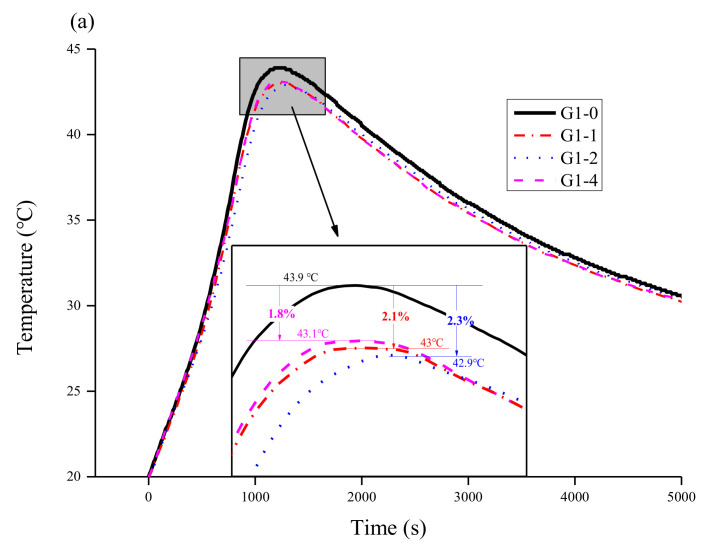

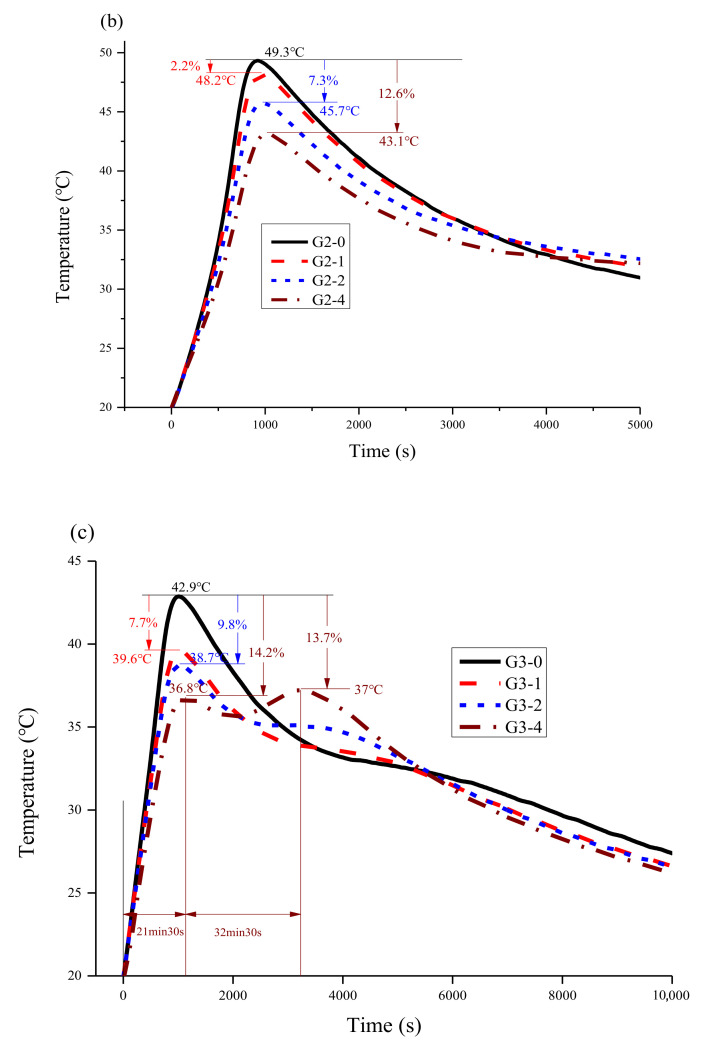
Hydration heat of the MKPC specimens as a function of glass powder content (0, 10, 20, and 40 wt.%) and M/P ratio of (**a**) 1:1, (**b**) 2:1, and (**c**) 3:1.

Figure 3 shows the temperature curve of the MKPC with different amounts of glass powder (0 wt.%, 10 wt.%, 20 wt.%, 40 wt.%) during curing at a room temperature of 20 ℃. Figure 3a shows the temperature curve of the sample with an M/P ratio of 1:1. G1-1, G1-2, and G1-4 decreased by 2.1%, 2.3%, and 2.1%, respectively, compared with the G1-0 peak temperature, and the time to reach the peak hydration temperature increased by 0%, 0.4%, and 0.9%, respectively. Figure 3b shows that G2-1, G2-2, and G2-4 decreased by 2.2%, 7.3%, and 12.6%, respectively, compared with the peak temperature of G2-0, and the time to reach the peak temperature increased by 2.7%, 2.9%, and 5.7%, respectively. These results indicate that the exothermic peak temperature of the MKPC matrix doped with glass powder decreased significantly with an increase in the amount of glass powder. The time required to reach the peak of G2-0 was prolonged with the increase in glass powder. In Figure 3c (M/P ratio = 3:1), G3-1, G3-2, and G3-4 were 7.7%, 9.8%, and 14.2% lower than that of G3-0, respectively. The time to reach the peak temperature increased by 0.1%, 0%, and 5.7% for G3-1, G3-2, and G3-4, respectively, and the peak temperature decreased more significantly than that of the samples with low M/P ratios. Notably, the temperature change curve at a high M/P ratio (3:1) showed a second exothermic peak before the glass powder content reached 40 wt.%. The addition of glass powder reduced the maximum exothermic temperature of hydration of the MKPC matrix and prolonged the time to reach the peak value of temperature.

The hydration reaction of MPC can be divided into six stages [32]: (1) the dissolution period of the phosphate, (2) the initial dissolution period of MgO, (3) the formation of various hydration intermediates in the hydration process (such as Mg(H_2_O)_6_^2+^, phosphorrösslerite (MgHPO_4_·7H_2_O), newberyite (MgHPO_4_·3H_2_O), and Mg_2_KH(PO_4_)_2_·15H_2_O; the reaction intermediates are formed at high water content and low M/P ratios, (4) the growth acceleration period of the main hydration product struvite-K, (5) the growth deceleration period of the main hydration product struvite-K, and (6) the final hydration stability period. The main exothermic stage occurs during (2), (4), and (5).

The dissolution of MgO and hydration of Mg^2+^ ions are given by the following formulas:

(1) $$\mathrm{MgO}+2H^{+}={\mathrm{Mg}}^{2+}+H_{2}O$$

(2) $${\mathrm{Mg}}^{2+}+6H_{2}O=\mathrm{Mg}{\left(H_{2}O\right)}_{6}^{2+}$$

Reaction equations for phosphatite (MgHPO_4_ 7H_2_O), newberyite (MgHPO_4_ 3H_2_O), and Mg_2_KH (PO_4_)_2_ ·15H_2_O.

(3) $$\mathrm{MgO}+H_{2}{\mathrm{PO}}_{4}^{-}+7H_{2}O={\mathrm{MgHPO}}_{4}\cdot 7H_{2}O+{\mathrm{OH}}^{-}$$

(4) $$\mathrm{MgO}+H_{2}{\mathrm{PO}}_{4}^{-}+3H_{2}O={\mathrm{MgHPO}}_{4}\cdot 3H_{2}O+{\mathrm{OH}}^{-}$$

(5) $$2\mathrm{MgO}+K^{+}+2H_{2}{\mathrm{PO}}_{4}^{-}+14H_{2}O\to \mathrm{MgKH}{\left({\mathrm{PO}}_{4}\right)}_{2}\cdot 15H_{2}O+{\mathrm{OH}}^{-}$$

Based on previous analysis results, the glass powder was added as a filler in the hydration stage. Glass powder weakens the interaction between raw materials due to the large ratio of length to width of the particles and their irregular, angular, and flaky morphology. Friction readily occurs between the particles, which hinders the binding force between the raw material and free water, thus reducing the overall exothermic hydration reaction. Since the heat release of the overall hydration reaction is reduced, the time to reach the peak temperature is prolonged [33]. In the exothermic hydration curve with a high M/P ratio (3:1), the addition of 40 wt.% glass powder induced a second exothermic peak that occurred during stages (4) and (5) of hydration, which is the formation stage of the main hydration product struvite-K. The reaction resulted from the combination of Mg(H_2_O)_6_^2+^, phosphate, and free water, but unreacted MgO was still present in the system. This hindered the combination of the three materials in this stage. The addition of glass powder reduced the content of unreacted MgO, minimizing the restriction between raw materials, which prolonged the formation stage of hydration products in (4) and (5), and increased the hydration reaction, leading to the appearance of the second exothermic peak.

### 3.3. Compressive Strength

**Figure 4:**
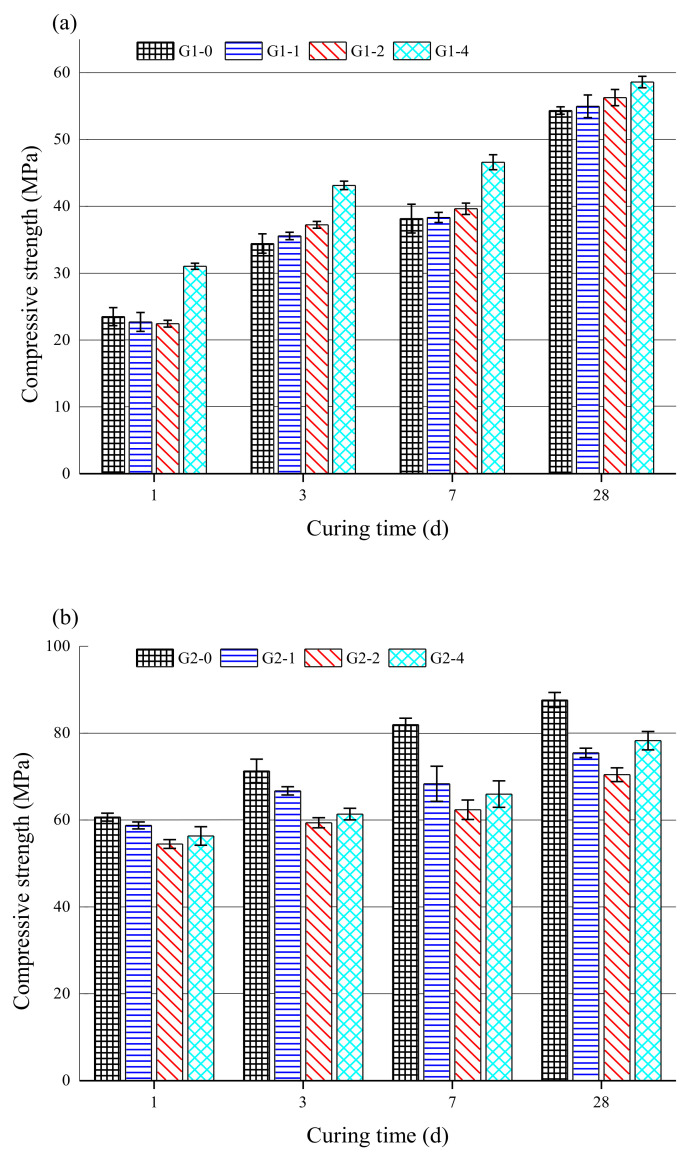

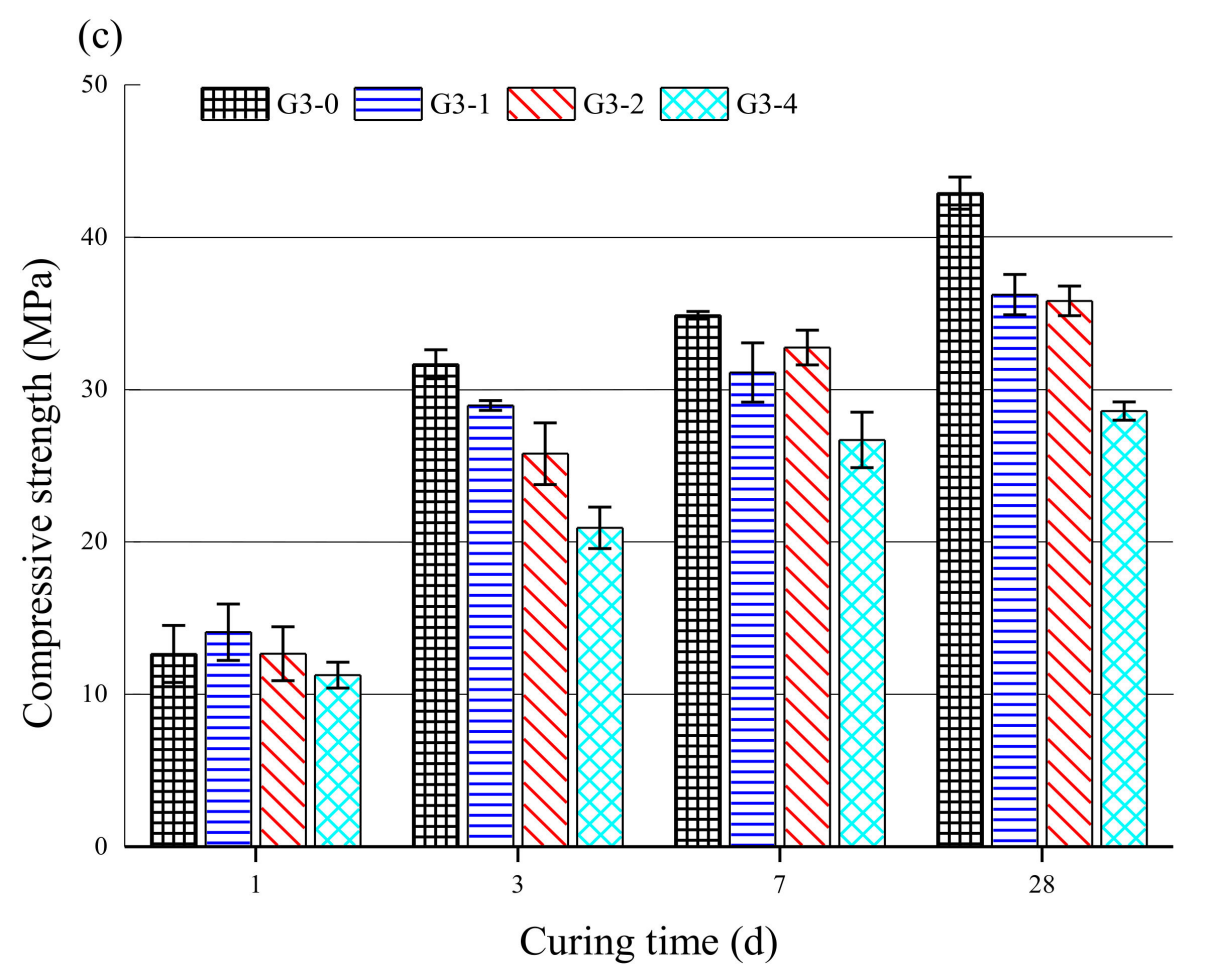
Compressive strength of the MKPC specimens with various glass powder contents (0, 10, 20, and 40 wt.%) and M/P ratios of (**a**) 1:1, (**b**) 2:1, and (**c**) 3:1.

The effect of the glass powder content on the compressive strength of the MKPC is shown in Figure 4, where Figure 4a–c represents the MKPC pastes with different M/P ratios of 1:1, 2:1, and 3:1, respectively. The results, shown in Figure 4a, indicate that the compressive strength of MKPC pastes increased gradually with an increase in the glass powder content. The compressive strength of G1-4 reached 58.6 MPa after curing for 28 d, which is about 7.9% higher than that of G1-0 without glass powder at the same curing age. The strength increase is related to the improved particle packing, because the glass powder filled the gaps between raw material particles, producing a denser structure [34]. In Figure 4b,c, the compressive strength of the MKPC pastes was reduced by adding glass powder. As shown in Figure 4b, the early compressive strength of G2-4 with 40 wt.% glass powder decreased by about 8.1% compared with G2-0. In Figure 4c, the compressive strength of the MKPC with a higher M/P ratio decreased significantly after the addition of glass powder, and the compressive strength of G3-4 was only 28.5 MPa at 28 d. Given that more unreacted MgO is associated with a high M/P ratio, replacing the MgO with 40 wt.% glass powder eliminated even more active magnesium oxide, which affected the hydration reaction and reduced the crystallinity of the hydration products, resulting in a decrease in compressive strength.

**Figure 5:**
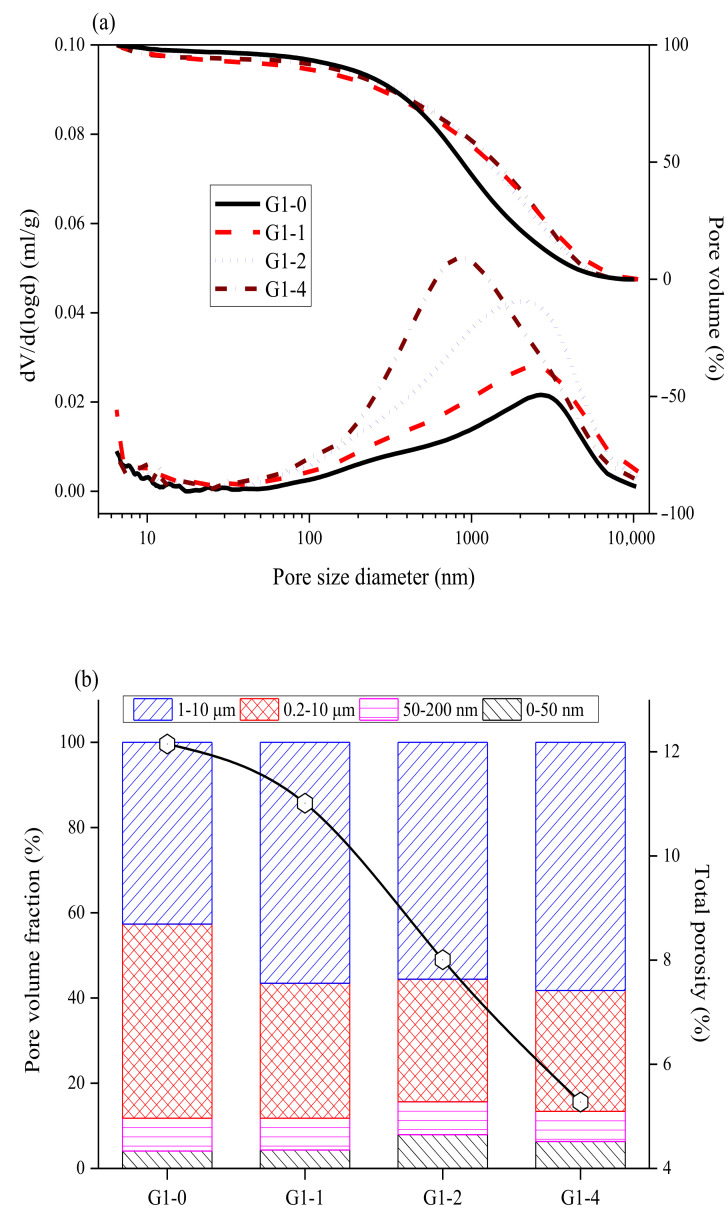
The MIP results for the hardened pastes at an M/P ratio of 1:1 after curing for 28 d: (**a**) pore size distribution and (**b**) total porosity and pore volume fraction.

In the mercury intrusion porosimetry (MIP) results shown in Figure 5, the porosity of the MKPC pastes with an M/P ratio of 1:1 decreased with an increase in the glass powder content. The pores in the G1-0 matrix shown in Figure 5a were mainly concentrated between 1000 and 10,000 nm. After the addition of the glass powder, the overall pore concentration interval shifted to the left, indicating that the addition of glass powder reduced the pore size of the MKPC matrix. Figure 5b shows the pore size distribution and overall porosity of the G1 group, where G1-0 had a total porosity of 12.1%. After adding the glass powder, the overall porosities of G1-1, G1-2, and G1-4 decreased to 11.0%, 8.0%, and 5.3%, respectively, indicating that glass powder reduced the porosity of the MKPC. The porosity was also closely related to the compressive strength. Reducing the porosity increased the overall density of the paste, thus improving its compressive strength. Therefore, the glass powder filled the interstices between the MKPC materials, thereby reducing the porosity of the MKPC paste.

### 3.4. Hydration Products

**Figure 6:**
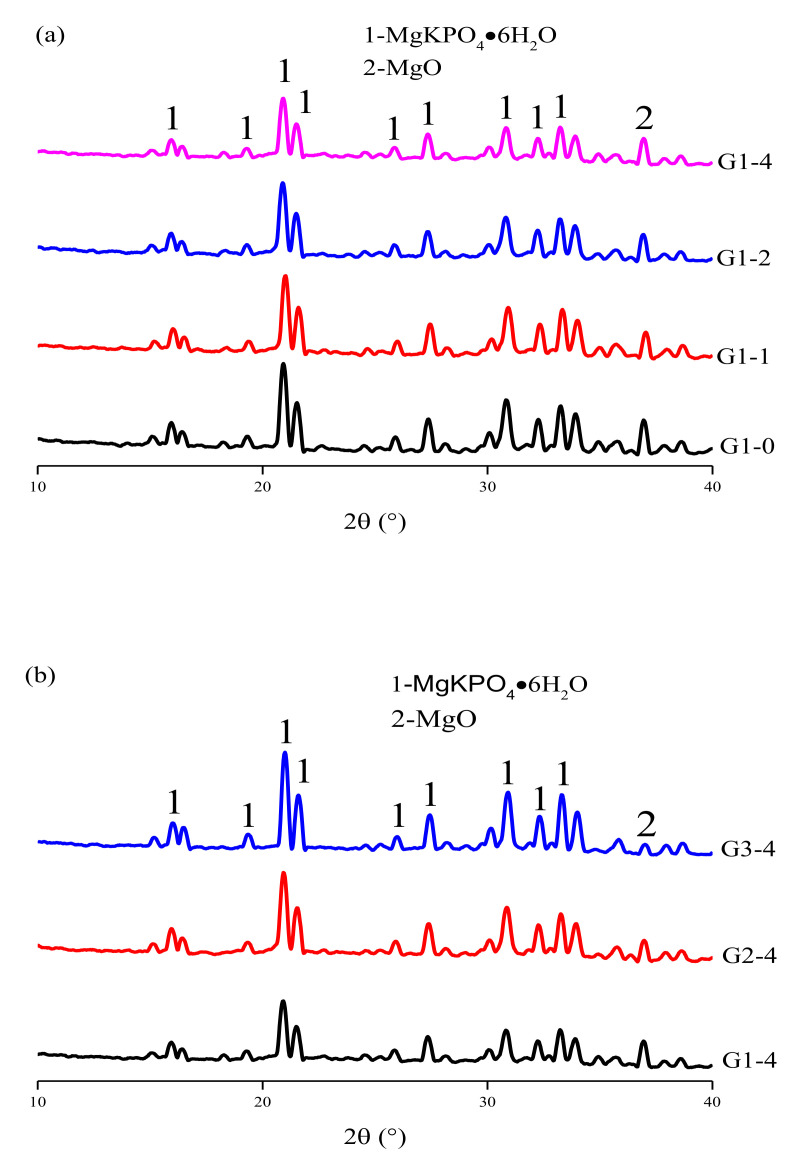
XRD patterns of the samples hydrated for 28 d: (**a**) samples with different glass powder concentrations and an M/P ratio of 3:1, and (**b**) samples with different M/P ratios and a glass powder concentration of 40 wt.%.

**Figure 7:**
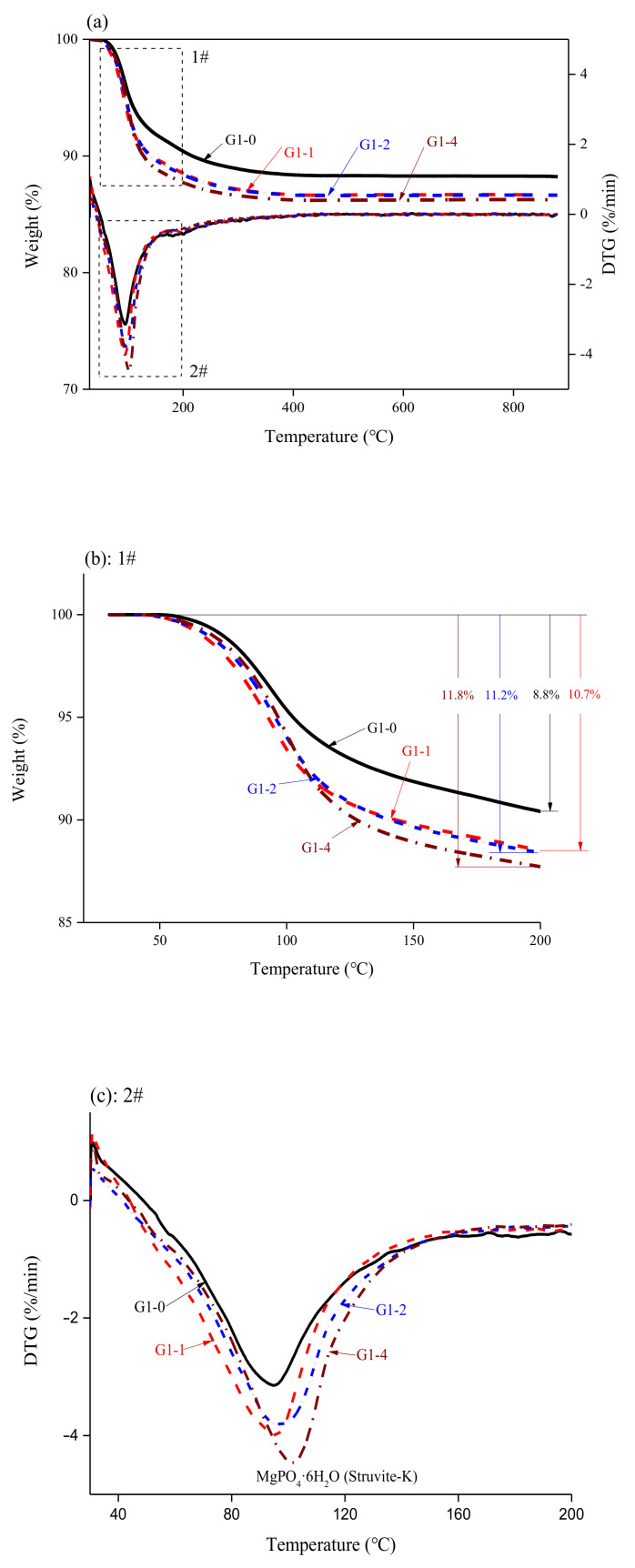
TG/DTG curves of the MKPC pastes with an M/P ratio of 1:1, containing 0 to 40 wt.% glass powder cured for 28 d, where (**a**) shows all curves and (**b**) and (**c**) are expanded graphs of areas 1 and 2 in (**a**).

Figure 6 shows XRD patterns for the hydration products of the samples. The primary phases were struvite-K and magnesia, and no other Ca-, or Si-related products were detected. However, it does not rule out the reaction between the glass powder and the cement matrix, because it is difficult for the XRD diffractometer to detect the substance with a content of less than 5 wt.% in the sample. In a study by Tao Zhang [35], when the M/P ratio was greater than 1.00, the end of hydration pH was 12.1, and the pH of the MPC was concentrated at 12–13. This indicates that, theoretically, the glass powder can react with alkaline substances in the later stage of hydration of an MKPC paste. The TG-DTG analysis of our hydration products is shown in Figure 7a. The primary weight loss of the MKPC slurry occurred in the range of 30–200 °C, due to the change in quality caused by dehydration of the main hydration product struvite-K [36]. The mass loss is shown in Figure 7b, indicating that, as the glass powder content increased at an M/P ratio of 1:1, the formation of hydration product struvite-K increased. The strength of MKPC is mainly attributed to the combination of the hydration product and reactive MgO. In this case, the main hydration product, struvite-K, was used as the binder, and unreacted MgO was used as the aggregate to fill the struvite-K structure. The increase in hydration products further densified the three-dimensional network structure in the later stages [37]. Furthermore, the glass powder filled some of the voids in the structure, and the overall porosity decreased.

**Figure 8:**
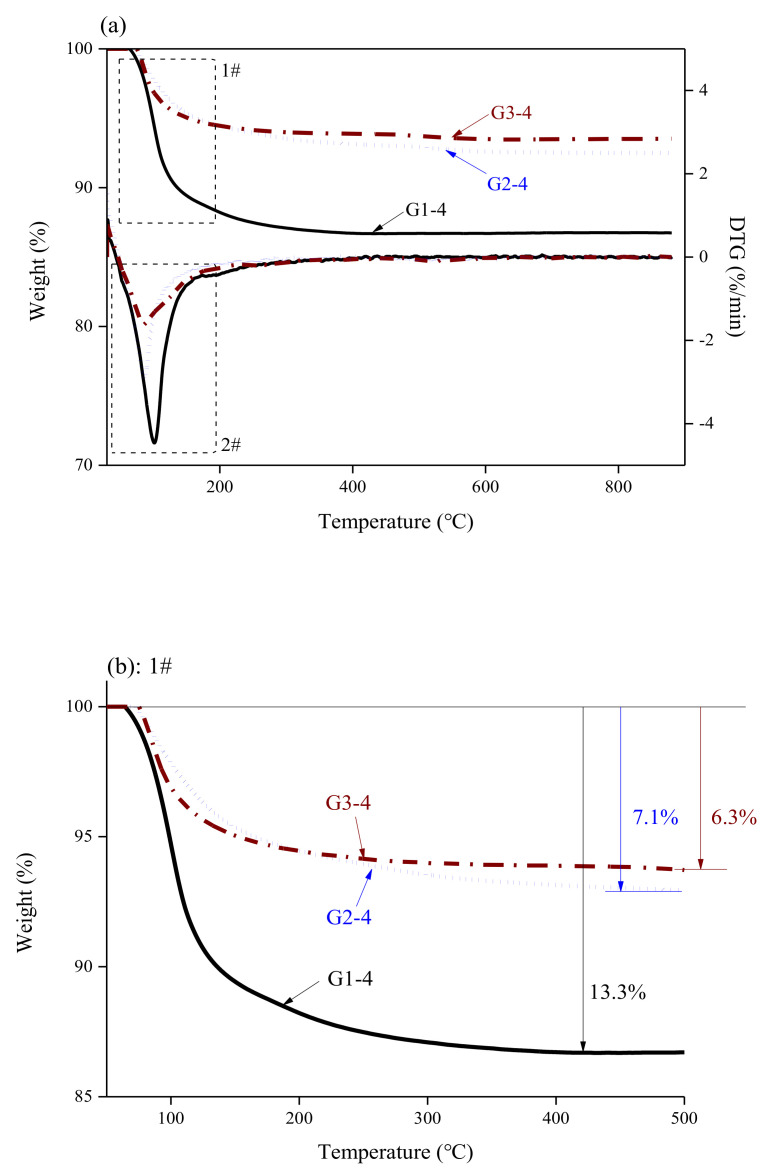

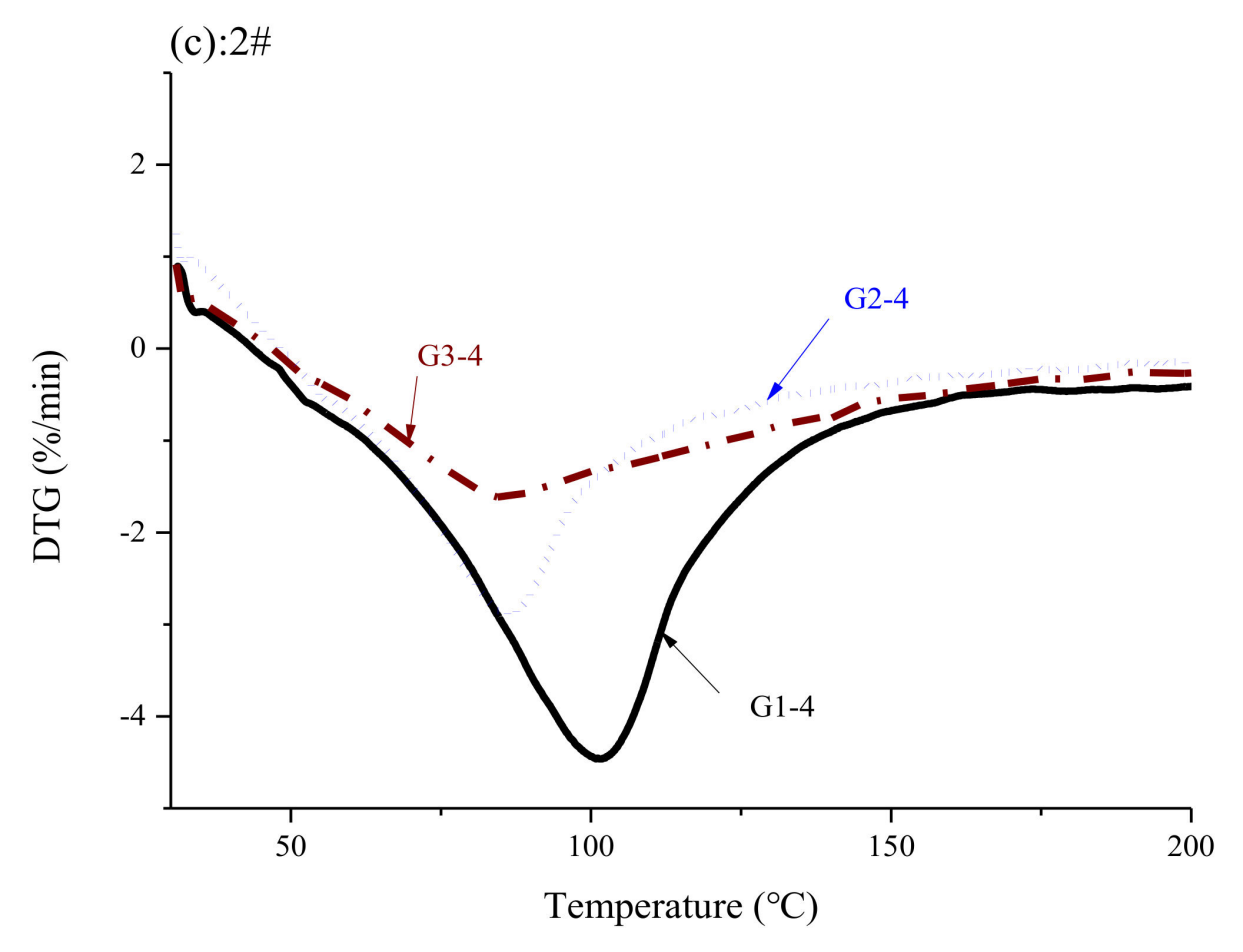
TG/DTG curves of samples cured for 28 d with different M/P ratios and 40 wt.% glass powder concentration, where (**a**) shows all curves and (**b**,**c**) are expanded graphs of areas 1 and 2 in (**a**).

Figure 8 shows the TG/DTG curves of the MKPC paste mixed with 40 wt.% glass powder under various M/P ratios. The curves indicate that the mass loss of the MKPC paste was reduced at higher M/P ratios for 40 wt.% glass powder additions. From room temperature to 900 °C, the total mass losses were 13.3%, 7.1%, and 6.3%, for G1-4, G2-4, and G3-4, respectively. Adding an appropriate amount of glass powder to MKPC at a lower M/P ratio increased the crystallization rate of MPC and increased the formation of struvite. However, under high M/P ratios, the active MgO content in the MKPC paste was higher, and the KH_2_PO_4_ content was lower than that of the low M/P ratio sample. Even if an appropriate amount of glass powder could have been added to speed up the reaction, the yield of K-struvite was restricted by the KH_2_PO_4_ content. GP can increase the crystallization rate of MPC and form more phases in MPC, including sodium phosphate and calcium phosphate gels [27]. Thus, adding an appropriate amount of glass powder to MKPC contributed to the sufficient growth of matrix strength.

### 3.5. Microstructural Analysis

**Figure 9:**
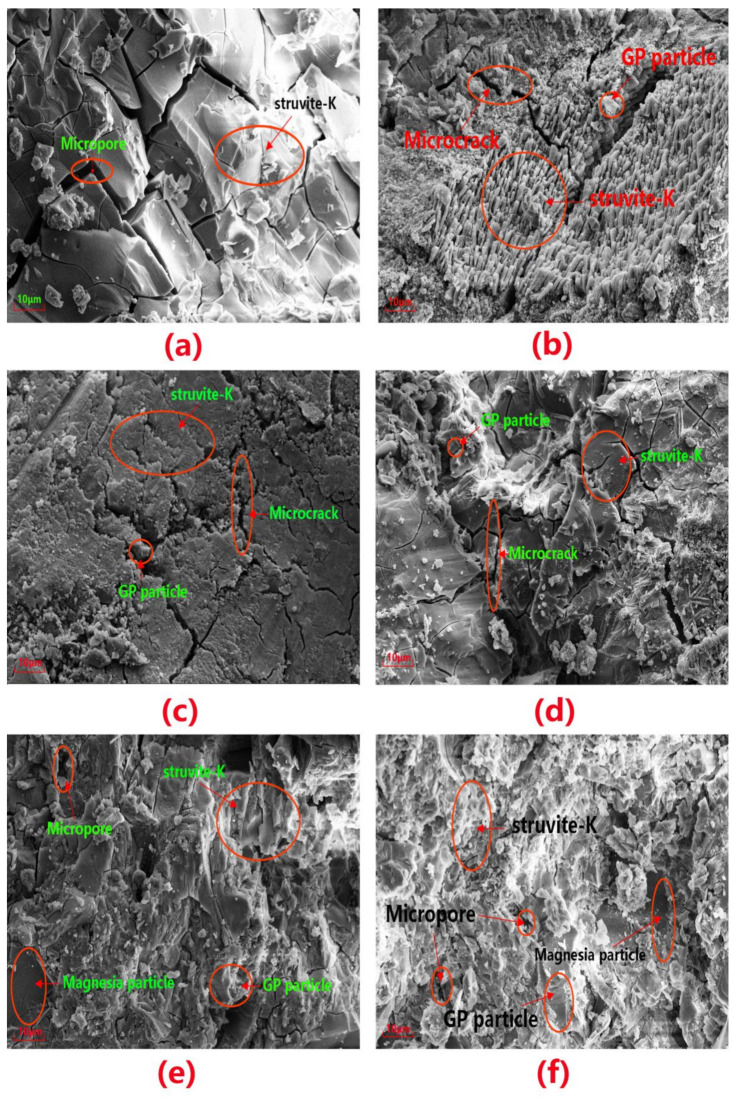
SEM images of the MKPC pastes cured for 28 days: (**a**) G1-0 (**b**) G1-1 (**c**) G1-2 (**d**) G1-4 (**e**) G2-4 and (**f**) G3-4.

Figure 9 shows the microstructure of the MKPC mortar after 28 d of curing. Figure 9a shows a typical morphology of struvite-K, the hydration product of MKPC paste without glass powder, which appears as blocky grains. Moreover, the paste without GP exhibited many and more numerous microcracks than those with GP. In Figure 9b,c, the hydration products of the MKPC paste doped with 10 wt.% and 20 wt.% glass powder exhibits a significant change in morphology compared to the paste without GP. In this case, the glass powder filled the voids between the struvite-K and enhanced the density of the matrix. The partial replacement of magnesia by GP increased the number of grains, potentially providing more sites for the hydration and crystallization processes in MPC [27]. The morphology of struvite-K gradually changed from blocks to short columns with higher glass contents, and the larger pores in the MKPC gradually transformed into smaller pores. The growth morphology of struvite-K mainly depends on its growth conditions (including growth space and M/P ratio) [38]. In Figure 9d, the addition of GP particles formed a small flat gel around the blocky struvite hydration product or embedded itself in the cracks of the product. This caused the micropores and microcracks in part of the MKPC matrix to disappear, making the matrix more compact.

As shown in Figure 9, with an M/P ratio of 1:1, the addition of GP partially formed a gel, which hindered the normal hydration process, resulting in a morphological transformation of hydration products. As GP content increased, peeling patches appeared on the surface of the matrix, caused by the spalling of unreacted GP and struvite-K blocks. The bonding ability between the hydration products and the MKPC decreased, and exfoliated patches covered the surface of the matrix, appearing as a mass of powder [39]. Many micropores were apparent on the surface of the MKPC matrix, and were even visible to the naked eye. The structural density further decreased as GP content increased, reducing the compressive strength of the MKPC paste.

### 3.6. Chemical Composition Analysis of the Micro-Regions

**Figure 10:**
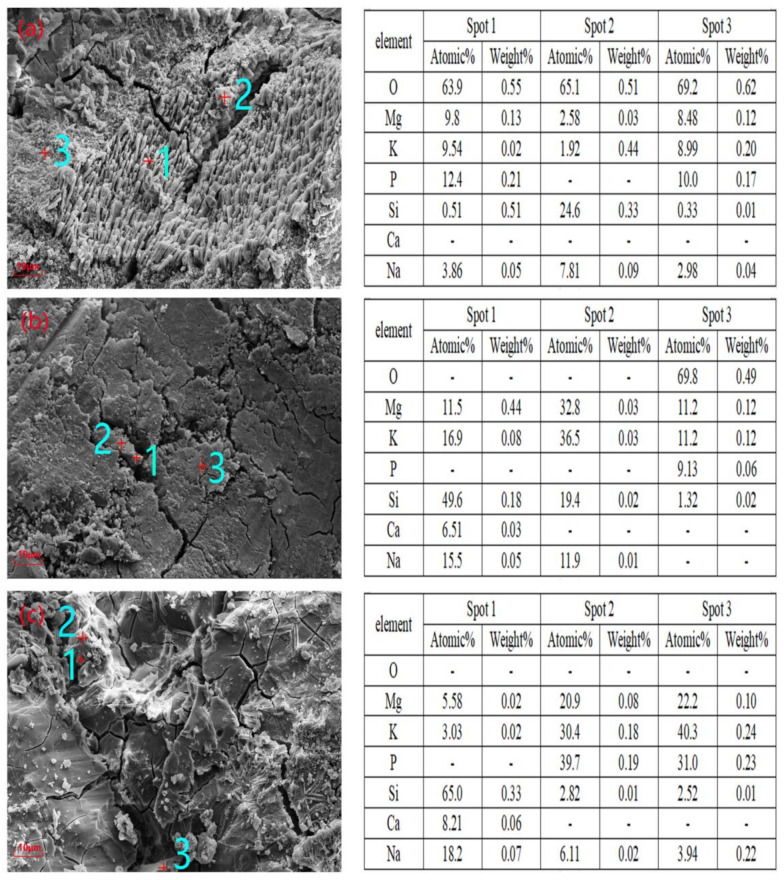
SEM-EDS analyses on spots of (**a**) G1-1, (**b**) G1-2, and (**c**) G1-4 cured for 28 days.

Figure 10 shows the SEM-EDS analysis results of G1-1, G1-2, and G1-4 after curing for 28 days, indicating the atomic concentration percentage and mass ratio of each element. In Figure 10a, spot 1 is a short columnar particle typical of struvite-K with an O:Mg:K:P:Si:Na mass ratio of 0.55:0.13:0.21:0.51:0.05, also indicative of the hydration product struvite-K. The mass ratio of the main elements O:Mg:K:Si:Na in spot 2 in Figure 10a was 0.51:0.03:0.44:0.33:0.09. Ground glass powder has a higher specific surface area than cement, so GP has a good pozzolanic effect [26]. This area is suspected to be an unreacted GP particle embedded on the hydration product surface, indicating that GP is involved in the hydration reaction in the matrix. At spot 3, the mass ratio was 0.62:0.12:0.20:0.17:0.01:0.04 for O:Mg:K:P:Si:Na, considered to be massive struvite. Thus, the addition of glass powder induced a change in the morphology of the hydration products. In Figure 10b, the mass ratio of spot 1 was 0.04:0.08:0.18: 0.03 for Mg:K:Si:Ca:Na, likely indicating that it is a component of sodium silicate in MPC. In addition, the mass ratio of the main elements of spots 2 and 3 suggests the presence of a hydration product from the hydration stage of MPC. Furthermore, the mass ratio of the square blocky grain of spot 1 in Figure 10c was 0.02:0.33:0.33:0.06:0.07 for Mg:K:Si:Ca:Na, and the mass ratio of the main elements of Mg:K:Si:Na in spot 2 was 0.08:0.18:0.19:0.01:0.02. Therefore, we believe that the GP particles were partially involved in the hydration reaction and attached to the hydration products.

**Figure 11:**
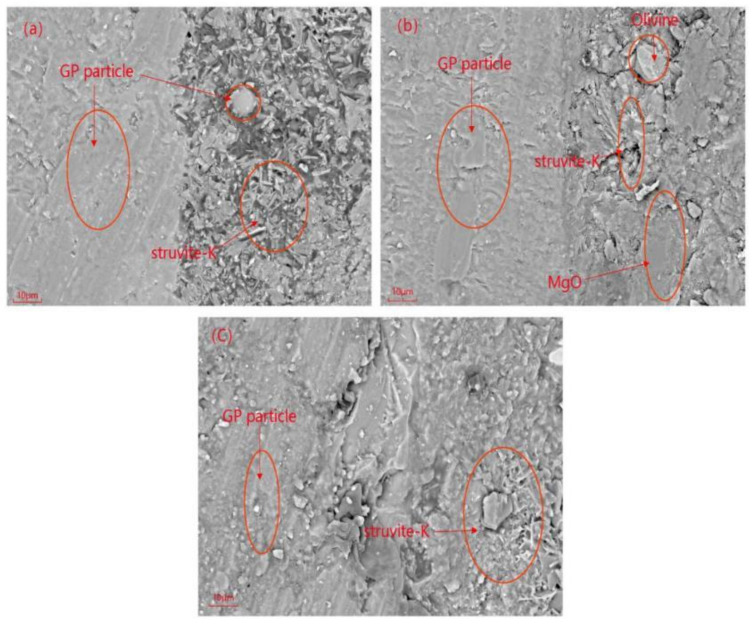
SEM images of the MPC pastes mixed with glass fragments: (**a**) M/P ratio = 1:1, 20%; (**b**) 2:1, 40%; (**c**) 3:1, 40%.

**Figure 12:**
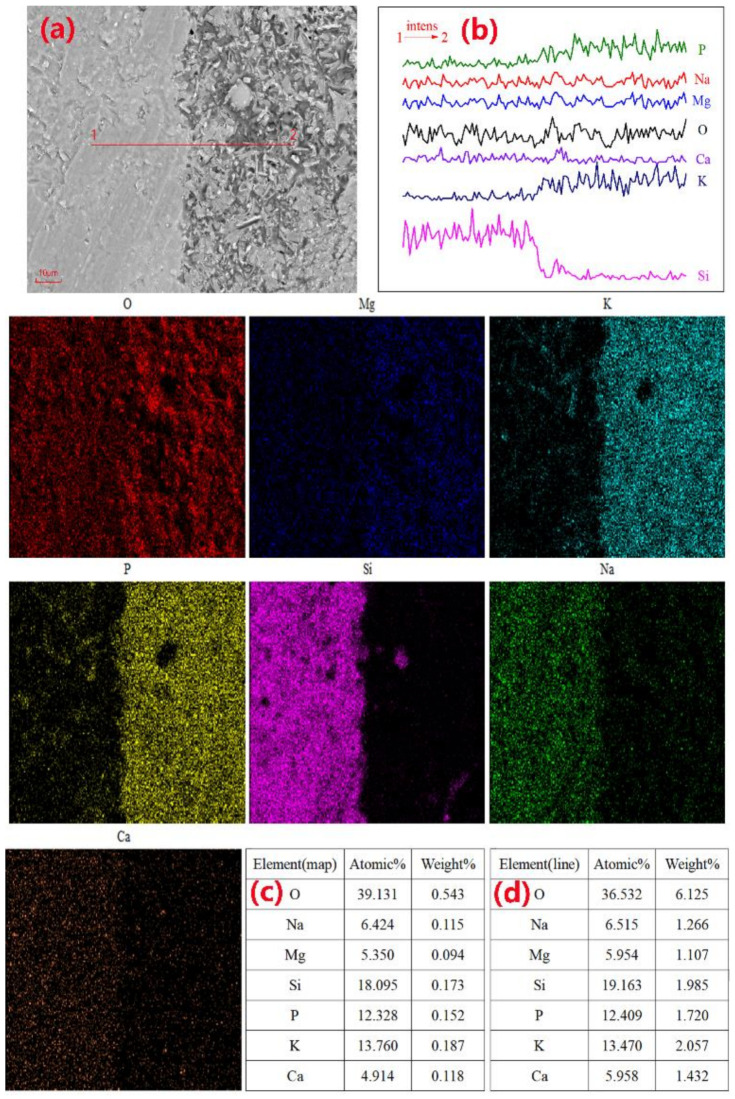
SEM images and elemental maps along lines 1–2 of the MKPC pastes cured after 7 days: M/P ratio = 1:1 and 40 wt.% content of glass fragments, (**a**) SEM image; (**b**) Intensity of each element distribution along line 1-2; (**c**) EDS elemental distribution along lines 1–2; (**d**) EDS elemental maps.

**Figure 13:**
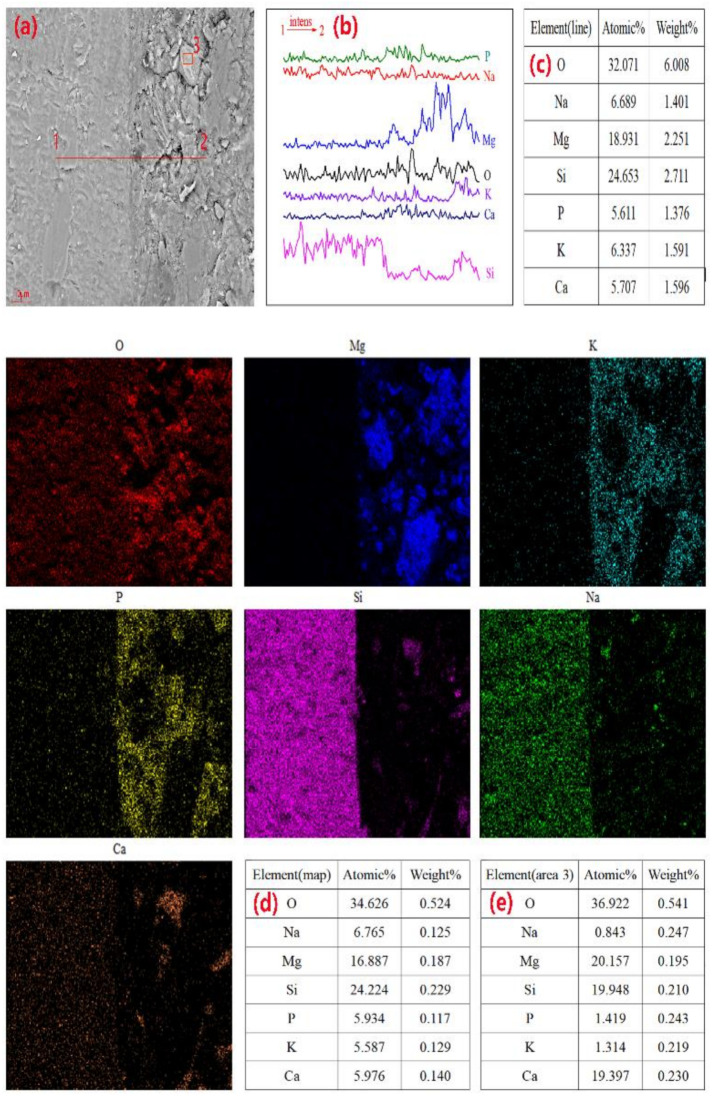
SEM images and element maps along lines 1–2 of the MKPC pastes cured after 7 days: M/P ratio = 2:1 and 40 wt.% content of glass fragments, (**a**) SEM image; (**b**) Intensity of each element distribution along line 1-2; (**c**) EDS elemental distribution along lines 1–2; (**d**) EDS elemental maps; (**e**) EDS elemental area 3.

**Figure 14:**
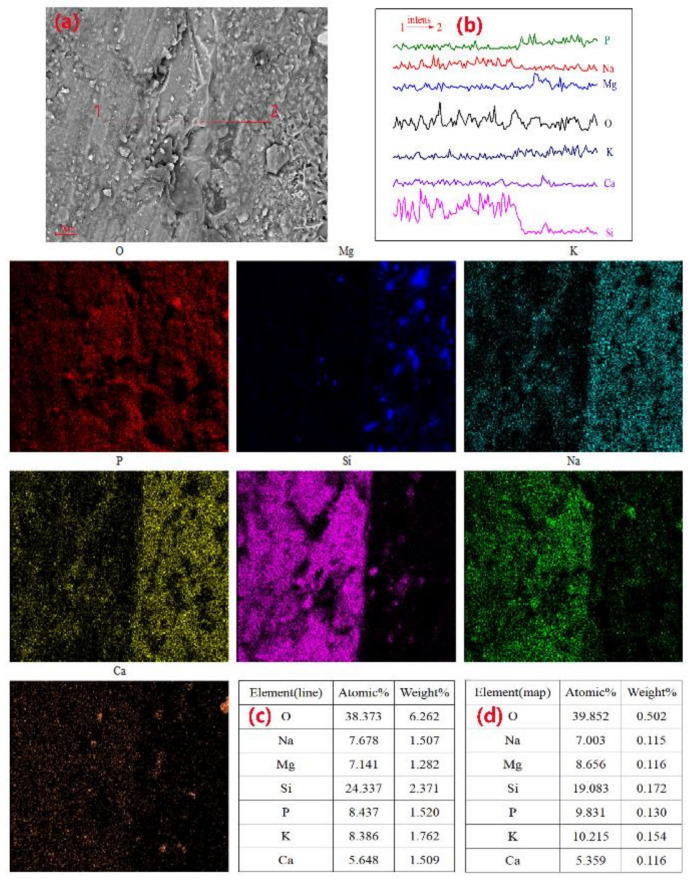
SEM images and element maps along lines 1–2 of the MKPC pastes cured after 7 days: M/P ratio = 3:1 and 40 wt.% content of glass fragments, (**a**) SEM image; (**b**) Intensity of each element distribution along line 1-2; (**c**) EDS elemental distribution along line 1-2; (**d**) EDS elemental maps.

To more intuitively observe the reaction of the GP particles in the MPC hydration stage, we conducted an experiment in which GP fragments with a particle size distribution of 0.1–1 mm were mixed into MPC paste. After 7 d of curing and subsequent SEM-EDS testing, the elemental distribution at the interface between the glass fragments and MPC paste was observed. When the M/P ratio was 1:1, the SEM image (Figure 11a) showed a close bond between the glass fragments and the MPC matrix. Figure 12 shows the distribution of the elements in the sample in Figure 11a. In Figure 11b,c, there are small cracks at the joint between the MKPC matrix and glass fragments, and the pores on the surface of the matrix become larger. During the line scan from point 1 to point 2, the intensities of Si, Na, and Ca in the glass phase ranged from high to low. The surface scan shows that the distribution of these three elements at the junction of the GP and MPC matrix was overlaid with the distribution of elements Mg, P, and K. Si was distributed between MPC matrices, indicating that a small part of the edge of the GP fragments was involved in the hydration process. Figure 13 and Figure 14 indicate that the excessive amount of MgO in the MPC matrix reduced the yield of hydration products with an increase in the M/P ratio, and struvite-K was partially coated on the surface of the glass fragments. In addition, overlapping Si, Mg, O, and Ca elemental patterns (area 3) were also observed in Figure 13. The elements identified in this test area are suspected to be a new phase composed of Ca, Mg, and Si elements. This indicates that the glass fragments were partially involved in the hydration reaction.

## 4. Conclusions

The effect of glass powder on the properties of MKPC was investigated, resulting in the following observations.

The addition of glass powder prolonged the setting time of MKPC. At an M/P ratio of 2:1, the setting time of MKPC blended with 40 wt.% glass powder was 11% longer than that of the sample without glass powder. The addition of glass powder also reduced the workability of the MKPC pastes. The reduction in workability increased with an increase in the M/P ratio and an increase in the glass powder content.

The addition of glass powder can effectively reduce the heat release during the hardening of MKPC. When the GP content and the M/P ratio increased, the peak reduction of the hydration heat of MKPC was significant.

When the M/P ratio was 1:1, the addition of glass powder made the MKPC structure dense and improved the compressive strength of the matrix. The analysis of the hydration products indicated that the alkali components in the GP participated in the reaction of MKPC, and the hydration products were closely bound to the MPC matrix. However, with the increase in the M/P ratio, the compressive strength of the MKPC structure decreased with increasing glass powder content. Under high M/P ratios, the increase in the GP content and the excess MgO occupied most of the void space in the MKPC, and some of the hydration products adhered to the surface of the GP, resulting in a decrease in the overall strength of the matrix.

## Figures and Tables

**Table 1:** Chemical composition of dead-burnt MgO and glass powder in wt.%.

Chemical Composition (wt.%)	Dead-Burned Magnesia	Glass Powder
MgO	94.3	3.52
Al_2_O_3_	0.4	0.99
Fe_2_O_3_	0.4	0.15
SiO_2_	3.1	70.76
CaO	1.7	9.71
Na_2_O	-	14.11
K_2_O	-	0.34
SO_3_	-	0.28
Others	0.1	0.12

**Table 2:** Mix proportion design of the sample.

Samples	M/P ^a^	Glass Powder (wt.%)	B/M ^b^ (wt.%)	w/b ^c^
G1-0	1:1	0	0.1	0.15
G1-1	1:1	10		
G1-2	1:1	20		
G1-4	1:1	40		
G2-0	2:1	0		
G2-1	2:1	10		
G2-2	2:1	20		
G2-4	2:1	40		
G3-0	3:1	0		
G3-1	3:1	10		
G3-2	3:1	20		
G3-4	3:1	40		

^a^ M/P is the MgO-KH_2_PO_4_ mass ratio; ^b^ B/M is the borax-MgO mass ratio; ^c^ w/b is the water-binder mass ratio.

## Data Availability

The datasets used and/or analyzed during the current study are available from the corresponding author on reasonable request.
